# Effect of *Stellaria media* Tea on Lipid Profile in Rats

**DOI:** 10.1155/2020/5109328

**Published:** 2020-01-24

**Authors:** Virág Demján, Tivadar Kiss, Andrea Siska, Márton Richárd Szabó, Márta Sárközy, Imre Földesi, Dezső Csupor, Tamás Csont

**Affiliations:** ^1^University of Szeged, Faculty of Medicine, Department of Biochemistry, Metabolic Diseases and Cell Signaling Group (MEDICS), 6720 Szeged, Dóm tér 9, Hungary; ^2^University of Szeged, Interdisciplinary Centre for Natural Products, 6720 Szeged, Eötvös utca 6, Hungary; ^3^University of Szeged, Interdisciplinary Centre of Excellence, 6720 Szeged, Dugonics tér 13, Hungary; ^4^University of Szeged, Faculty of Pharmacy, Department of Pharmacognosy, 6720 Szeged, Eötvös utca 6, Hungary; ^5^University of Szeged, Faculty of Medicine, Department of Laboratory Medicine, 6725 Szeged, Semmelweis utca 6, Hungary

## Abstract

**Background:**

In folk medicine, common chickweed (*Stellaria media*) has traditionally been applied for the treatment of hypercholesterolemia; however, there is no firm experimental proof to support the rationale of this practice. Therefore, we aimed to assess the efficacy and safety of *Stellaria media*) has traditionally been applied for the treatment of hypercholesterolemia; however, there is no firm experimental proof to support the rationale of this practice. Therefore, we aimed to assess the efficacy and safety of *Materials and Methods*. Adult male Wistar rats were divided into 3 groups. The (i) control group received standard laboratory chow, the (ii) hypercholesterolemic group received cholesterol-enriched diet, and the (iii) chickweed-treated hypercholesterolemic group received cholesterol-enriched diet and 100 mg/kg body weight *Stellaria media*) has traditionally been applied for the treatment of hypercholesterolemia; however, there is no firm experimental proof to support the rationale of this practice. Therefore, we aimed to assess the efficacy and safety of

**Results:**

Cholesterol-enriched diet significantly increased serum total cholesterol, LDL- and HDL-cholesterol levels, but did not affect triacylglycerol concentrations. The addition of chickweed to the diet did not cause any significant change in serum lipid profile or body weight increase. Liver and kidney functions were unaltered and cardiac morphology and function were not changed due to *Stellaria media*) has traditionally been applied for the treatment of hypercholesterolemia; however, there is no firm experimental proof to support the rationale of this practice. Therefore, we aimed to assess the efficacy and safety of

**Conclusion:**

Although chickweed does not seem to be toxic, our results do not support the rationale of its use in the treatment of hypercholesterolemia.

## 1. Introduction

The wide ecological tolerance and short-term vegetative reproduction period make chickweed (*Stellaria media* (L.) Vill., Caryophyllaceae) a common and widespread species. In cool, moist, and moderately shaded environment, huge territories are covered by this plant; thus, its presence in cultivated fields is a serious agricultural problem.

Although chickweed has been consumed as salad and has been applied in folk medicine as tea [1, 2], its safety and efficacy have not been investigated in clinical trials [3]. Moderate interest has been shown toward this plant from the middle of the last century. Because of the potential biological benefits and its application in cosmetics, phytochemical and pharmacological studies have started to focus on species of *Stellaria* genus. These studies are based mainly on *in vitro* or *in vivo* animal experiments. According to these studies, several *Stellaria* species have noteworthy pharmacological activities (e.g., antibacterial, anti-inflammatory, and antiallergic effects) [4].

Nowadays, *Stellaria media*, mostly consumed as tea, is gaining popularity as a remedy to lose weight [5] and it is widely used for its believed beneficial effects on blood lipid profile [6]. According to a popular Hungarian traditional healer, the infusion of 2.5 g chickweed two times daily normalizes increased cholesterol level within some weeks [7]. Moreover, consumption of chickweed tea for cholesterol lowering is recommended by several websites dealing with health and lifestyle issues [8, 9]. Since there is no clinical evidence supporting this hypothesis and the designs of the available animal experiments differ from the human use, the aim of the present work was the investigation of chickweed tea on rats to gather additional data on efficacy and safety.

## 2. Methods and Materials

### 2.1. Animals

The experiment conforms to the Guide for the Care and Use of Laboratory Animals published by the U.S. National Institutes of Health (National Institutes of Health publication 85–23, revised 1996), and the regulations of the Hungarian Act No. XXVIII of the year 1998 on protection and care of animals were strictly followed. The study was approved by the local animal ethics committee of the University of Szeged (XV.1181/2013).

Altogether 24 adult (8-week old) male Wistar rats were used in this study (purchased from Charles River Laboratories, Göttingen, Germany), weighing 270–324 g at the onset of the experiments. Using only male rats in the study was intentional as the hormonal changes during the menstrual cycle of females have been shown to influence serum lipids [10, 11]. Rats were kept under standard climatic conditions (22 ± 2°C room temperature, 12 h light/dark cycles), in pairs, in individually ventilated cages (Sealsafe IVC system, Buguggiate, Italy) and had *ad libitum* access to tap water and laboratory rat chow.

### 2.2. Experimental Setup

After one week of acclimatization, the rats were randomized into three groups: control (Cont), hypercholesterolemia (HChol), and hypercholesterolemia + *Stellaria media* treatment (HChol + SM). Rats in the control group (*n* = 8) received standard laboratory rat chow. The other 16 rats were fed a special cholesterol-enriched diet, i.e., a standard laboratory rat chow (Innovo Ltd., Isaszeg, Hungary) supplemented with 2% (w/w) cholesterol (Hungaropharma, Budapest, Hungary) and 0.25% (w/w) sodium-cholate-hydrate (Sigma-Aldrich, St. Louis, USA) for 8 weeks to induce experimental hypercholesterolemia as described earlier [12–14]. We have chosen this cost-effective model of experimental hypercholesterolemia because our research group has previously accumulated extensive experience regarding the use of this model [12–14] and the lipoprotein profile of the cholesterol-fed rats (LDL/HDL ratio) is quite similar to that of humans. The diet of 8 animals receiving cholesterol-enriched chow was further supplemented with *Stellaria media* tea lyophilizate mixed into cookie balls (HChol + SM) in order to examine the potential cholesterol-lowering effect of *Stellaria media*. On the eighth week, rats were anesthetized with sodium pentobarbital (Euthasol, 50 mg/kg body weight, ip., Produlab Pharma b.v., Raamsdonksveer, The Netherlands), the abdominal cavity was opened, and blood samples were taken from the abdominal aorta. Collected blood was allowed to clot and was centrifuged (2000 × g, 20 min, 4°C); then serum was separated for analysis of various serum parameters to evaluate the efficacy and safety of *Stellaria media* treatment, including lipid profile and parameters representing liver and kidney function. Before termination, echocardiography was performed in order to evaluate the effects of experimental hypercholesterolemia and *Stellaria media* on cardiac morphology and function.

### 2.3. Preparation of *Stellaria media* Tea Lyophilizate

*Stellaria media* was harvested in Algyő (Hungary) by “Ezerjófű” Association in 2017. Voucher specimen (no: 882) was deposited in the herbarium of the University of Szeged, Faculty of Pharmacy, Department of Pharmacognosy. The drug was dried and stored at room temperature.

The dried and grounded drug was extracted with boiling water (1 : 10 w/v ratio) for 15 minutes by ultrasonication. The highly dense extract was separated from solid particles by mechanical press, and the water extract was lyophilized. Approximately, 1.5 g lyophilizate was obtained from 10.0 g dried drug.

### 2.4. *Stellaria media* Administration

Rats in the HChol + SM group received 100 mg/kg body weight lyophilized *Stellaria media* tea mixed into cookie balls once a day. The recipe of cookie dough included 55% plain flour, 20% caster sugar, and 25% water [15]. All animals received 2 g cookie dough/kg body weight per day. We have found in a pilot study that administration of 2 g/kg body weight cookie dough for 7 days in control rats did not cause significant changes in levels of blood cholesterol, triacylglycerol, or glucose (data not shown). The dough was prepared once a week and kept at 4°C until use. The 100 mg/kg body weight dose of lyophilized *Stellaria media* tea was considered as equal to human daily dose, calculated according to Nair and Jacob [16]. Individual portions of lyophilized *Stellaria media* tea were freshly mixed with the cookie balls right before administration. During the one-week long acclimatization period, the rats were habituated to the cookie balls in order to prevent neophobia and were trained to accept the cookie balls voluntarily in their home cages. We always made sure that the whole cookie ball was eaten, and the success rate of this technique was 100% during the experiment. Cookie balls were preferred instead of the traditional gavage technique in order to cause less daily stress to the animals and to model human exposure the most objectively.

### 2.5. Measurements of Serum Lipid Levels

Serum total cholesterol, high-density lipoprotein (HDL) cholesterol, triacylglycerol levels, and pancreatic lipase enzyme activities were analyzed by using Roche Cobas 8000 analyzer system in the Department of Laboratory Medicine using enzymatic colorimetric assays from Roche (Mannheim, Germany). Low-density lipoprotein (LDL) cholesterol levels were measured using a kit from Diagnosticum, Budapest, Hungary, adopted to a plate reader (FLUOstar Optima, BMG), as described earlier [17].

### 2.6. Measurements of Serum Parameters Representing Liver and Kidney Function

Several other serum parameters were measured using Roche Cobas 8000 analyzer system to monitor the effect of diet-induced hypercholesterolemia as well as *Stellaria media* treatment on liver and kidney functions. Total protein, albumin, and creatinine concentrations as well as alkaline phosphatase (ALP) enzyme activities were analyzed by colorimetric assays from Roche (Mannheim, Germany). Alanine aminotransferase (ALT) and aspartate aminotransferase (AST) enzyme activities and carbamide levels were measured with Roche UV assays (Mannheim, Germany), as described earlier [14].

### 2.7. Transthoracic Echocardiography

Cardiac morphology and function were assessed by transthoracic echocardiography at week 8 as described previously [18–20]. Briefly, rats were anesthetized with sodium pentobarbital (Euthasol, 50 mg/kg body weight ip.). Then, the chest was shaved and the rat was placed in a supine position onto a heating pad. Two-dimensional and M-mode echocardiographic examinations were performed by the criteria of the American Society of Echocardiography with a Vivid IQ ultrasound system (General Electric Medical Systems, Boston, USA) using a phased array 5.0–11 MHz transducer (GE 12S-RS probe). Data of three consecutive heart cycles were analyzed (EchoPac Dimension software; General Electric Medical Systems, Boston, USA) by an experienced investigator in a blinded manner. The mean values of three measurements were calculated and used for statistical evaluation.

### 2.8. Statistical Analysis

Statistical analysis was performed by using SigmaPlot 12.0 for Windows (Systat Software Inc). All values are presented as mean ± SEM. One-way ANOVA was used to determine the differences among the three experimental groups. *p* < 0.05 was accepted as statistically significant difference, using the Tukey *post hoc* test.

## 3. Results

### 3.1. Body Weight

**Figure 1:**
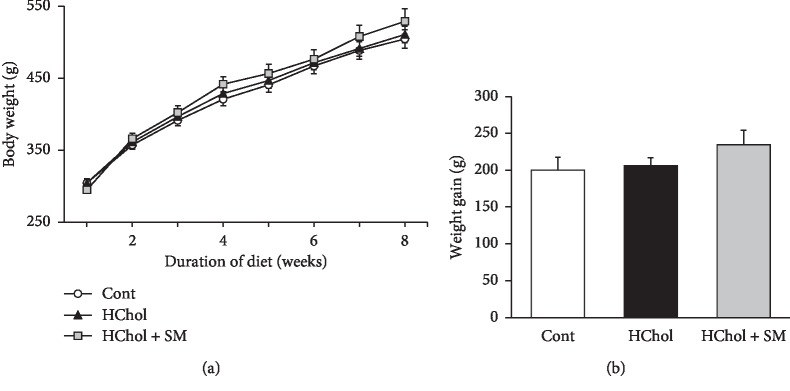
Body weight (a) and weight gain after 8 weeks (b) in the control group (white spheres) and rats fed with cholesterol-enriched diet (black triangles) or cholesterol-enriched diet with *Stellaria media* extract (grey squares). Results are means ± SEM (*n* = 8 in each group), analyzed by one-way ANOVA with Tukey *post hoc* test.

Body weight showed a continuous increase from 305 ± 4 g at the onset of the experiment to 505 ± 13 g at week 8 in the control group fed a normal diet (Figure 1(a)). Neither cholesterol-enriched diet nor *Stellaria media* treatment affected body weight significantly at any time points (Figure 1(a)). Weight gain during the 8-week feeding protocol was also not affected significantly by any of the treatments (Figure 1(b)).

### 3.2. Serum Lipid Parameters

**Figure 2:**
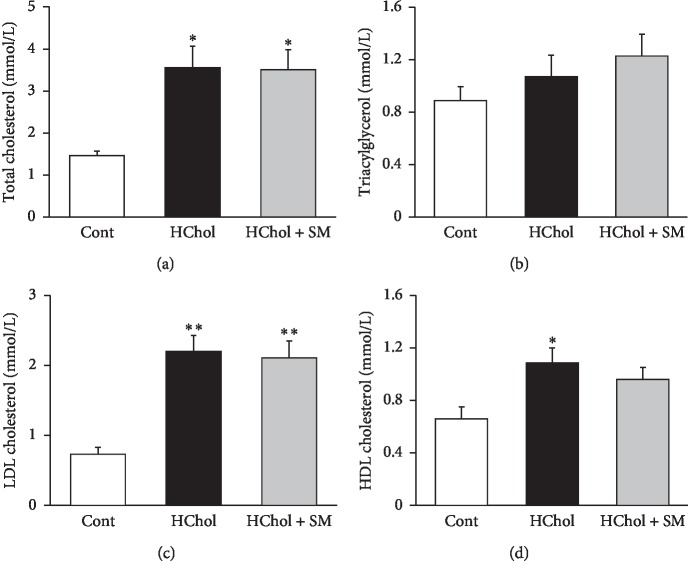
Serum total cholesterol (a), serum triacylglycerol (b), LDL cholesterol (c), and HDL cholesterol (d) levels in rats fed either cholesterol-enriched (HChol), cholesterol-enriched + *Stellaria media* (HChol + SM), or normal diet (Cont). Results are means ± SEM (*n* = 8 in each group), analyzed by one-way ANOVA with the Tukey *post hoc* test. ^*∗*^*p* < 0.05 vs. control, ^*∗∗*^*p* < 0.01 vs. control.

Lipid levels were measured from serum in order to validate the development of diet-induced hypercholesterolemia by the end of an 8-week feeding protocol. Total cholesterol concentration was significantly elevated in the HChol and the HChol + SM groups compared to the control group; however, there was no significant difference between HChol and HChol + SM values (Figure 2(a)). Triacylglycerol levels showed no significant difference due to cholesterol-enriched diet or *Stellaria media* treatment (Figure 2(b)). Similarly to total cholesterol, serum LDL cholesterol concentration was significantly higher in the HChol group, which was not affected by *Stellaria media* tea lyophilizate (Figure 2(c)). Serum HDL cholesterol level was significantly higher in the HChol group compared to control values; however, *Stellaria media* treatment did not affect significantly HDL cholesterol level (Figure 2(d)). Serum pancreatic lipase enzyme activities were not statistically different among the three experimental groups (Cont: 5.5 ± 0.27 U/L, HChol: 7.25 ± 1.07 U/L, and HChol + SM: 6.13 ± 0.35 U/L). These results suggest that *Stellaria media* tea lyophilizate does not have triacylglycerol- and cholesterol-lowering effect.

### 3.3. Liver Weight and Function

**Figure 3:**
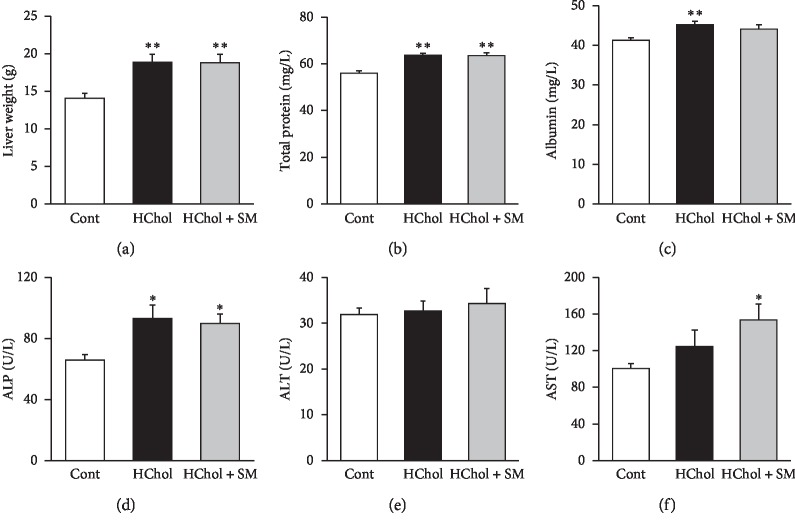
Liver weight (a), serum total protein levels (b), albumin levels (c), alkaline phosphatase (ALP) activity (d), alanine aminotransferase (ALT) activity (e), and aspartate aminotransferase (AST) activity (f) in rats fed either cholesterol-enriched (HChol), cholesterol-enriched + *Stellaria media* (HChol + SM), or normal diet (Cont). Results are means ± SEM (*n* = 8 in each group), analyzed by one-way ANOVA with the Tukey *post hoc* test. ^*∗*^*p* < 0.05 vs. control, ^*∗∗*^*p* < 0.01 vs. control.

Diet-induced hypercholesterolemia caused marked alterations in some liver parameters. Liver weight, serum total protein, and albumin concentrations, as well as ALP activity were significantly higher in the HChol group compared to the control group (Figures 3(a)–3(d)). ALT and AST activities were not altered due to diet-induced hypercholesterolemia (Figures 3(e) and 3(f)). *Stellaria media* treatment did not influence significantly the hypercholesterolemia-induced alterations (Figure 3). Interestingly, AST enzyme activities were significantly higher in the HChol + SM group compared with the control group.

### 3.4. Kidney Function

**Figure 4:**
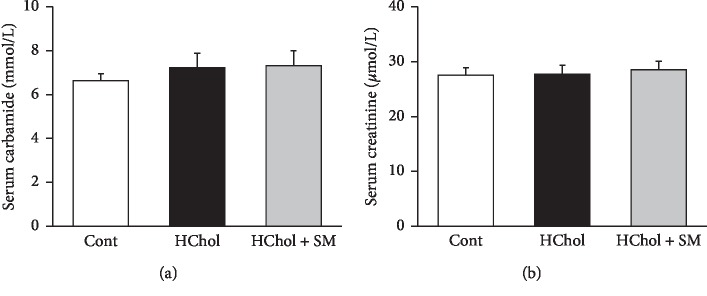
Serum carbamide (a) and creatinine (b) levels in rats fed either cholesterol-enriched (HChol), cholesterol-enriched + *Stellaria media* (HChol + SM), or normal diet (Cont). Results are means ± SEM (*n* = 8 in each group), analyzed by one-way ANOVA with the Tukey *post hoc* test.

Diet-induced hypercholesterolemia and *Stellaria media* treatment did not influence the serum parameters representing kidney function since there was no significant difference among the experimental groups in serum carbamide and creatinine levels (Figure 4).

### 3.5. Transthoracic Echocardiography

Transthoracic echocardiographic measurements performed at the end of the feeding protocol showed that diet-induced hypercholesterolemia and *Stellaria media* treatment did not affect cardiac morphology as there were no significant differences in systolic and diastolic wall thickness parameters (Table 1). Parameters related to cardiac function including left ventricular end-diastolic and end-systolic volume, stroke volume, ejection fraction, and heart rate were also not influenced significantly by diet-induced hypercholesterolemia and *Stellaria media* treatment (Table 1).

## 4. Discussion

In folk medicine, *Stellaria media* is mostly consumed as tea and is believed to decrease blood cholesterol level. In accordance, several tea products are available with this claim or indication. Nevertheless, the cholesterol-lowering effect of *Stellaria media* tea has not been investigated previously.

In our present study, we intended to model the human use of chickweed as close as possible. For this reason, according to folk medicinal practice, only above-ground parts of *Stellaria media* were used. The extract was prepared as tea infusion like in human use, and the dosage was calculated according to the typical human dose. Since we were primarily interested in the potential cholesterol-lowering effect of *Stellaria media*, we applied an experimental model of diet-induced hypercholesterolemia that has been previously characterised and extensively used in our laboratory [12–14]. In our present study, we did not confirm the cholesterol-lowering effect of *Stellaria media* tea. No alterations in lipid metabolism were observed since pancreatic lipase activity was not inhibited, and blood lipid profile (i.e., total cholesterol, triacylglycerol, LDL, and HDL cholesterol) was not significantly different compared to the untreated hypercholesterolemic group.

In the literature, the antiobesity effect of *Stellaria media* was examined using various rodent obesity models [21–23]. Only one of these studies demonstrated a cholesterol-lowering effect of *Stellaria media* administered as 900 mg/kg body weight lyophilized juice in a high-fat diet-induced obesity model in male Swiss albino mice [23]. In the same study, the lyophilized juice of *Stellaria media* also reduced the high-fat diet-induced increase in triacylglycerol level and body weight. Nevertheless, lyophilized juice at 400 mg/kg body weight had no beneficial effects. The contradictions of these findings with our results are likely due to significant differences in the experimental setups. The major difference is the type and dose of *Stellaria media* extracts used in the studies. We treated animals with *Stellaria media* tea lyophilizate at a dose of 100 mg/kg body weight, while the other research group used lyophilized herb juice at an effective dose of 900 mg/kg body weight. The different extraction methods likely resulted in qualitative and quantitative differences in the active substances of the extracts, and the dose of the lyophilized juice of *Stellaria media* seems to be unrealistically high.

In two other publications, ethanolic and methanolic extracts of *Stellaria media* were tested in progesterone-induced obesity [22] or in cafeteria diet-induced obesity models in female rats or mice [21]. Administration of 400 mg/kg body weight methanolic extract decreased triacylglycerol levels in both obesity models, but it did not affect total cholesterol levels. The ethanolic extract had no beneficial effects in these studies. It is worth mentioning that the administration of methanolic extract has no relevance in human use and the assessment of the ethnopharmacological application of chickweed. Unfortunately, credibility of the data in these latter two papers is rather questionable because there are numerous contradictions between the reported data in the tables and the description and interpretation of the findings in the text. Furthermore, the results are not comparable with the findings of other reports since the poorly described extraction method is unclear.

We have also investigated some safety issues, and based on our results, SM treatment does not seem to have a severe toxic effect on the liver or the kidneys, since several liver marker enzymes were elevated in the HChol group without being affected by SM treatment (total protein, albumin, and ALP). Although AST enzyme activities were elevated in the HChol + SM group compared with the control group, the AST activity in the HChol + SM group did not differ from the values measured in the HChol group. Elevated AST level in the blood is often considered as a sign of liver damage; however, AST is not specific for the liver and may be also increased due to injuries of the heart, muscle, pancreas, kidney, or red blood cells [24]. Interestingly, SM treatment actually was found to be hepatoprotective in a liver toxicity model [25]. Overall these data suggest that *Stellaria media* treatment has no deleterious effect on liver function; however, a possible limitation of our study is the lack of a group receiving herbal treatment without cholesterol-enriched chow.

## 5. Conclusions

In our current study, we have also investigated some safety issues and found that *Stellaria media* was neither toxic nor caused alterations in liver or kidney functions and cardiac morphology compared with hypercholesterolemic rats. This suggests a safe use of *Stellaria media* tea. The human use of chickweed tea for lowering blood cholesterol level was examined *in vivo* in rats, using an experimental design to mimic the human use of the herb. Since the body weight and blood lipid profile were not significantly altered in the group treated with *Stellaria media* compared with the group fed with cholesterol-enriched diet only, our experiment does not support the rationale for using chickweed tea in order to lower cholesterol level.

## Figures and Tables

**Table 1:** Effects of *Stellaria media* on left ventricular morphological and functional parameters. Transthoracic echocardiographic measurement values in rats fed either cholesterol-enriched (HChol), cholesterol-enriched + *Stellaria media* (HChol + SM), or normal diet (Cont). Results are means ± SEM (*n* = 8 in each group), analyzed by one-way ANOVA with the Tukey *post hoc* test. 2D: two dimensional; 4CH: four chambers view; MM: M (motion) Mode; ns: not significant.

Parameter (unit)	View/mode	Week 8			Significance
		Cont	HChol	HChol + SM	
Anterior wall thickness-systolic (mm)	Short axis/MM	3.57 ± 0.11	3.41 ± 0.17	3.10 ± 0.12	ns
Anterior wall thickness-diastolic (mm)	Short axis/MM	2.20 ± 0.11	2.22 ± 0.17	2.16 ± 0.11	ns
Inferior wall thickness-systolic (mm)	Short axis/MM	3.85 ± 0.11	3.46 ± 0.15	3.69 ± 0.09	ns
Inferior wall thickness-diastolic (mm)	Short axis/MM	2.14 ± 0.09	2.13 ± 0.14	2.19 ± 0.13	ns

Posterior wall thickness-systolic (mm)	Long axis/MM	3.82 ± 0.08	3.68 ± 0.12	3.62 ± 0.09	ns
Posterior wall thickness-diastolic (mm)	Long axis/MM	2.19 ± 0.03	2.39 ± 0.13	2.52 ± 0.19	ns
Septal wall thickness-systolic (mm)	Long axis/MM	3.79 ± 0.06	3.62 ± 0.12	3.54 ± 0.17	ns
Septal wall thickness-diastolic (mm)	Long axis/MM	2.50 ± 0.11	2.24 ± 0.10	2.35 ± 0.11	ns

Left ventricular end-diastolic volume (*μ*l)	4CH/2D	127 ± 15	129 ± 13	105 ± 13	ns
Left ventricular end-systolic volume (*μ*l)	4CH/2D	49 ± 8	53 ± 5	46 ± 6	ns
Stroke volume (*μ*l)	4CH/2D	79 ± 8	76 ± 8	59 ± 8	ns
Ejection fraction (%)	4CH/2D	63 ± 2	59 ± 1	56 ± 3	ns
Heart rate (1/min)	4CH/2D	343 ± 14	367 ± 8	370 ± 11	ns

## Data Availability

The data used to support the findings of this study are provided in this paper. Any further data are available from the corresponding author upon request.

## Abbreviations

2D: Two dimensional
4CH: Four-chamber view
ALP: Alkaline phosphatase
ALT: Alanine aminotransferase
AST: Aspartate aminotransferase
Cont: Control
HChol: Hypercholesterolemia
HChol + SM: Hypercholesterolemia + *Stellaria media*
HDL: High-density lipoprotein cholesterol
LDL: Low-density lipoprotein cholesterol
MM: M (motion) mode.
